# Proliferative diabetic retinopathy in patients with type 2 diabetes correlates with the presence of atherosclerosis cardiovascular disease

**DOI:** 10.1186/s13098-021-00666-z

**Published:** 2021-04-26

**Authors:** Lu Gao, Wei Zhao, Jin-Kui Yang, Ming-Zhao Qin

**Affiliations:** 1 Department of Geriatrics, Beijing Tongren Hospital, Capital Medical University, Beijing, 100730 China; 2 Department of Endocrinology, Beijing Tongren Hospital, Capital Medical University, Beijing, China

## Abstract

**Background:**

Atherosclerosis cardiovascular disease (ASCVD) is the main cause of morbidity and mortality in type 2 diabetes mellitus (T2DM). As most diabetic patients with ASCVD are asymptomatic, it is most neglected in clinical practice. For this reason, identifying high-risk ASCVD population with intensified treatment is very important. In recent years, the relationship between diabetic retinopathy (DR) and ASCVD has caused much academic concern, but the results are inconsistent. Moreover, whether all grades of DR increase the risk of ASCVD remains controversial. Most importantly, very few data can be found in China.

**Objective:**

Our aim is to discuss whether all grades of DR increase the risk of ASCVD after adjustment for the traditional cardiovascular risk factors and to assess the independent contribution of DR to cardiovascular events in patients with T2DM, hoping to provide more evidence for early identification of ASCVD.

**Research design and methods:**

A total of 425 T2DM patients with complete physical and biochemical data were included in the study. The grade of DR was assessed with two 45 color digital retinal images. Based on the presence of history of ASCVD, 425 T2DM patients were divided into 2 groups: ASCVD group and non-ASCVD group.

**Results:**

ASCVD patients were older and had a significantly higher fasting plasma glucose (FPG) and glycated haemoglobin (HbA1c) and proportion of history of ASCVD. At the same time, they were more likely to be females, and had lower level of alcohol and calculated glomerular filtration rate (eGFR) than non-ASCVD patients. Their trend to develop DR with ASCVD was significantly higher than patients with non-ASCVD (χ^2^ = 5.805, P  = 0.016). DR was an independent statistical indicator of the presence of ASCVD [odds ratio (OR) (95% CI): 2.321 (1.152–4.678), P = 0.018]. Furthermore, when DR was divided into non-proliferative retinopathy (NPDR) and proliferative retinopathy (PDR) according to its severity, only PDR was significantly associated with incident ASCVD [OR (95% CI): 8.333 (1.813–38.304), P = 0.006]. After adjusting for traditional ASCVD risk factors, such an association still existed [OR (95% CI): 7.466 (1.355–41.137), P = 0.021].

**Conclusion:**

DR associates strongly with ASCVD in the Chinese population with T2DM. With the increasing severity of DR, the risk of ASCVD also increases. After adjustment for traditional risk factors, PDR is still an independent risk marker for ASCVD.

## Introduction

Diabetic retinopathy (DR) is one of the most common and severe microvascular complications of diabetes mellitus (DM). WHO studies have found that the number of diabetic patients on a global scale reached 366 million in 2011, and this figure is projected to increase even further to over 500 million by the year 2025, and approximately one in three of those sufferers will develop DR [1]. Mounting DR severity has a positive correlation with vision impairment or loss as well as other vision-threatening proliferative diseases [2].

Defined by raised glycemic levels, DM is a major contributor to atherosclerosis cardiovascular disease (ASCVD), the leading cause of death among adults with diabetes mellitus [3]. For this reason, DM is widely seen as an ASCVD risk equivalent. Likewise, of all contributing factors of mortality and morbidity among the DM population, ASCVD tops the list, making up 70% of all deaths [4, 5]. However, DM patients are more likely to suffer from silent myocardial ischemia and myocardial infarction (MI), as up to one-third of those without any manifestation of chest pain experience an acute MI [6]. Worse still, these patients often fail to be treated timely and effectively in cases of emergency, which leads to soaring fatality rates. As a result, early recognition of such high-risk ASCVD population is of top importance.

The association between DR and ASCVD in patients with type 2 DM (T2DM) has drawn considerable international attention, as traditional risk factors for ASCVD, such as hypertension, hyperlipidemia, duration of hyperglycemia and magnitude of glycemia control are well known for the progression and development of DR [7]. There have been a great number of pathophysiological mechanisms that underlie ASCVD in diabetes, but few have been studied [8–13]. However, similar pathophysiological processes may contribute to ASCVD and DR. Previous research indicated that the presence of DR increases the risk of ASCVD, such as stroke [14, 15] and coronary heart disease (CHD) [16, 17]. However, other studies [18, 19] showed that this correlation subsides after adjustment for traditional ASCVD risk factors. What remains disputed is whether each stage of DR is associated with increased risk of ASCVD. Furthermore, present studies focus mainly on European and American people, leading to limited data on Asian people, especially Chinese. The aim of this study is to discuss whether the presence or severity of DR is associated with ASCVD independent of traditional cardiovascular risk factors and to evaluate the independent effect of DR on cardiovascular events in patients with T2DM.

## Research design and methods

### Study population

Participants of this study were diagnosed T2DM patients from geriatric and endocrine wards in Beijing Tongren Hospital, chosen between January 2018 and June 2020. 447 participants aged 50 or above who were willing to participate and subsequently signed an informed consent received examinations including a physical examination, blood glucose and lipid measurements, renal function tests and ophthalmic examination. After excluding subjects with cataracts, glaucoma, age-related retinopathy or other eye diseases, impaired renal function [calculated glomerular filtration rate (eGFR) < 60 ml/min], cancer, chronic obstructive pulmonary disease (COPD), T1DM, secondary diabetes and chronic pancreatitis, 425 participants with T2DM were divided into two groups (65 with ASCVD and 360 without) for a follow-up comparative study.

### Data collection

All participants received a standardized examination. Data regarding age, smoking, alcohol consumption, physical activity, educational level, and history of diabetes, hypertension and ASCVD were obtained with detailed medical records. Body mass index (BMI) was calculated as weight (kg) divided by height squared (m^2^). Waist circumference (WC) was measured at the level of the umbilicus in cm. Blood pressure (BP) was measured 3 times when participants were seated, and the average of the last 2 measurements was adopted. Blood samples were collected after an overnight fast for the determination of plasma glucose, HbA1c, total cholesterol (TC), triglycerides (TG), high density lipoprotein cholesterol (HDL-C), low density lipoprotein cholesterol (LDL-C) concentrations and serum creatinine (Scr). GFR was estimated by using the modified MDRD formula for Chinese patients [20]: eGFR (ml/min/1.73m^2^) = 175 × Scr^−1.234^ × age^−0.179^ × 0.79 (if female).

### Definition of clinical and biochemical variables

Various non-fatal ASCVD were determined according to a patient’s self-report. CHD can be defined with a history of myocardial infarction, at least one coronary stenosis more than 50% by coronary angiography or coronary CTA, a surgical history of revascularization (including percutaneous coronary intervention (PCI) or coronary artery bypass grafting (CABG)). The definition of stroke included a history of language or physical dysfunction continuing for more than 24 h and ischemic or hemorrhagic stroke diagnosed using imaging examination [computed tomography or magnetic resonance imaging]. ASCVD referred to a history of CHD or stroke, as defined above. Diagnosis of hypertension was based on meeting any of three criteria: systolic blood pressure (SBP) of ≥ 140 mmHg, diastolic blood pressure (DBP) of ≥ 90 mmHg or current use of antihypertensive drugs [21]. Central obesity was based on WC cutoff ≥ 80 cm in women and ≥ 90 cm in men according to the IDF ethnicity-specific definitions for Asian [22]. Overweight and obesity were respectively identified as BMI 24–28 kg/m^2^ and BMI equal to or over 28 kg/m^2^ according to the Working Group on Obesity in China (WGOC) (2002) [23]. Smokers were defined as those who had smoked ≥ 1 cigarette/day for at least 1 year. Drinkers were defined as those who had consumed ≥ 30 g of alcohol/week on average for at least 1 year. Regular leisure-time physical activity was defined as participation in moderate or vigorous activity for 30 min or more per day at least 3 days a week. Educational level was also recorded and categorized into two groups: low (illiteracy, primary, and secondary education), and high (high school education, college or university education). The family history of ASCVD went for first-degree relatives (biological mother, father, brothers, or sisters).

### Assessment of retinopathy

All participants received eye examinations by an ophthalmologist and had a bilateral retinal photograph taken of the fundus through dilated pupils. Two 45u color digital images of the retina were taken of each eye by a technologist using a Topcon TRC-NW7SF fundus camera (Topcon, Tokyo, Japan), an ophthalmic digital imaging system. The first image was centered on the macula, and the second on the optic nerve. The photographs were graded by two qualified ophthalmologists from the Eye Center of Capital Medical University, Beijing Tongren Hospital according to the international clinical diabetic retinopathy severity scale [24]: (i) no diabetic retinopathic changes (NDR); (ii) mild non-proliferative diabetic retinopathy (NPDR); (iii) moderate NPDR; (iv) severe NPDR; and (v) proliferative diabetic retinopathy (PDR). The degree of DR was determined according to the grading in the most affected eye. The ophthalmologists grading the photographs were blinded to the patients’ characteristics.

### Statistical analysis

All statistical analyses were conducted with the software package SPSS version 22.0 for Windows. For the continuous variables with a normal distribution, mean ± SD was reported and the independent t-test was used to compare subjects with no diabetic retinopathy (NDR) with any stage of diabetic retinopathy (DR). For the discrete variables or the continuous variables without a normal distribution, the median (P25–P75) was reported, and a Mann–Whitney rank test was used to examine the differences between the groups. In the meantime, distribution of discrete/qualitative variables was compared by Pearson chi-square test. Multivariable logistic regression analysis was used to estimate crude and adjusted odds ratios (ORs) (95% CIs) to allow for differences between groups with respect to demographic and risk factors and control for potentially confounding variables. A p-value of less than 0.05 was considered statistically significant.

## Results

A total of 425 T2DM patients with (n = 65) or without (n = 360) ASCVD comprised the study groups. There were 165 men and 260 women, with an average age of 58.72 ± 6.82. The total prevalence of DR was 11.29%, 9.65% with NPDR and 1.64% with PDR respectively.

The demographic and biochemical parameters of the two groups are shown in Table 1. Compared with the non-ASCVD group, patients with ASCVD were older, with higher levels of FPG and HbA1c and higher proportion of female and family history of ASCVD, while their level of eGFR and proportion of alcohol were lower. At the same time, there were no statistical differences between the two groups in duration of diabetes, the proportion of smoking, high educational level, physical activity, central obesity, generalized obesity, hypertension and the levels of SBP, DBP, TC, TG, HDL-C, LDL-C (Table 1).

**Table 1:** Clinical characteristic of studied subjects with and without ASCVD

	Total	Non-ASCVD	ASCVD	P value
Number (%)	425	360 (84.71)	65 (15.29)	
Age (years)	58.72 ± 6.82	58.27 ± 6.68	61.21 ± 7.12	0.001
Female (%)	260 (61.2)	212 (58.9)	48 (73.8)	0.023
Smoking, n (%)	112 (26.4)	101 (28.1)	11 (16.9)	0.061
Alcohol, n (%)	125 (29.4)	114 (31.7)	11 (16.9)	0.016
High educational level, n (%)	26 (6.1)	22 (6.1)	4 (6.2)	0.989
Physical activity, n (%)	332 (78.1)	281 (78.1)	51 (78.5)	0.942
Family history of ASCVD, n (%)	187 (44.0)	150 (41.7)	37 (56.9)	0.023
Central obesity, n (%)	311 (73.2)	257 (71.4)	54 (83.1)	0.050
General obesity				0.890
Overweight, n (%)	191 (44.9)	162 (45.0)	29 (44.6)	
Obesity, n (%)	132 (31.1)	113 (31.4)	19 (29.2)	
Duration of DM (years)	10.39 ± 3.24	10.34 ± 3.16	10.69 ± 3.70	0.416
FPG (mmol/l)	8.61 ± 2.64	8.50 ± 2.58	9.22 ± 2.95	0.042
HbA1c (%)	7.36 ± 1.75	7.25 ± 1.68	7.94 ± 2.04	0.004
Hypertension, n (%)	328 (77.2)	275 (76.4)	53 (81.5)	0.363
SBP (mmHg)	150.56 ± 20.17	150.30 ± 20.20	152.06 ± 20.11	0.518
DBP (mmHg)	87.16 ± 11.19	87.37 ± 11.25	85.98 ± 10.86	0.360
TC (mmol/l)	5.37 ± 1.14	5.37 ± 1.13	5.37 ± 1.19	0.977
TG (mmol/l)	2.31 ± 1.96	2.33 ± 2.05	2.19 ± 1.36	0.588
LDL-C (mmol/l)	2.71 ± 0.54	2.71 ± 0.53	2.73 ± 0.59	0.802
HDL-C (mmol/l)	1.45 ± 0.29	1.45 ± 0.29	1.47 ± 0.32	0.589
eGFR (ml/min/1.73 m^2^)	92.42 ± 17.68	93.31 ± 17.73	87.53 ± 16.69	0.015

Data are means ± SE or raw numbers (%). Continuous data were used for univariate general linear models and categorical data were analyzed by χ^2^ tests.

**Fig. 1:**
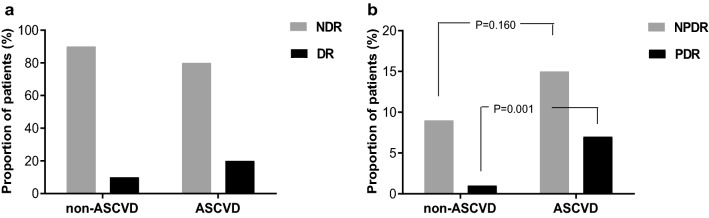
The association of ASCVD with DR or different degree of DR. **a** Comparison of prevalence of DR between ASCVD and non-ASCVD group. The trend to DR in the ASCVD group was significantly higher than in the non-ASCVD group (Pearson χ^2^ = 5.805, P = 0.016). **b** Prevalence (%) of patients with different degrees of DR (NPDR and PDR) in relation to ASCVD. When DR was divided into NPDR and PDR according to its severity, the prevalence of NPDR between ASCVD and non-ASCVD group had no statistical difference (Pearson χ^2^ = 1.975, P = 0.160). However, a higher proportion of subjects with PDR were found in the ASCVD group than from non-ASCVD group (Pearson χ^2^ = 10.368, P = 0.001)

Of the 360 subjects without ASCVD, 35 (9.7%) had DR. However, 13 cases (20.0%) were registered in the ASCVD group of 65 subjects, showing that the trend to DR in ASCVD group was significantly higher than in non-ASCVD group (Pearson χ^2^ = 5.805, P = 0.016), as is illustrated in Fig. 1a. Furthermore, when DR was divided into NPDR and PDR based on its severity, a higher proportion of subjects with NPDR (9, 13.8%) and PDR (4, 6.2%) were found in the ASCVD group than from non-ASCVD group [32 subjects (9.0%) with NPDR (Pearson χ^2^ = 1.975, P = 0.160) and 3 subjects (0.9%) with PDR (Pearson χ^2^ = 10.368, P = 0.001)], and the difference of prevalence of PDR in two groups was statistically significant (Fig. 1b).

**Fig. 2:**
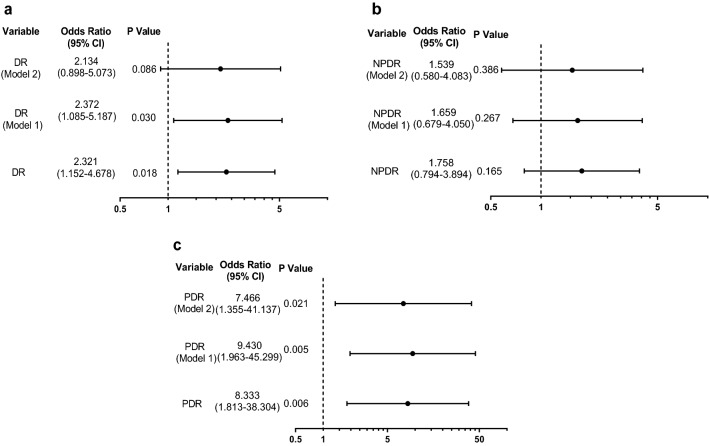
Odds ratios for ASCVD with/without DR and different degree of DR. Binary logistic regression was conducted to assess the association of ASCVD with DR (**a**) and different degrees of DR: NPDR (**b**) and PDR (**c**) using the Entry method; adjusted odds ratios (ORs) and the 95% confidence intervals (CIs) given. Adjustment variables included the basic confounders (age, sex, family history of ASCVD and duration of DM) in Model 1. In Model 2, smoking, alcohol, educational level, physical activity, obesity (central or general obesity), history of hypertension, SBP, DBP, HbA1c, LDL-C and eGFR were also considered other adjustment variables and were thus added to Model 1

Logistic regression analysis was performed to identify the risk factors of ASCVD. Age [odds ratio (OR) (95% CI): 1.814 (1.257–2.616), P = 0.001], female sex [OR (95% CI): 1.971 (1.091–3.562), P = 0.025], family history of ASCVD [OR (95% CI): 1.850 (1.085–3.155), P = 0.024] and HbA1c [OR (95% CI): 1.198 (1.050–1.368), P = 0.007] were relative risk factors and alcohol [OR (95% CI): 0.440 (0.222–0.872), P = 0.019], eGFR [OR (95% CI): 0.990 (0.827–0.980), P = 0.015] was relative protection factors of ASCVD (Table 2). Next, we found that DR [OR (95% CI): 2.321 (1.152–4.678), P = 0.018] was significantly associated with ASCVD, and such a link was only true for PDR [OR (95% CI): 8.333 (1.813–38.304), P = 0.006] when DR was divided into NPDR and PDR. With an adjustment for age, sex, family history of ASCVD and duration of DM, PDR was significantly associated with and an independent risk factor for ASCVD [OR (95% CI): 9.430 (1.963–45.299), P = 0.005]. Moreover, the association was not affected by an additional adjustment for smoking, alcohol, educational level, physical activity, obesity (central or general obesity), history of hypertension, SBP, DBP, HbA1c, LDL-C and eGFR [OR (95% CI): 7.466 (1.355–41.137), P = 0.021] (Fig. 2).

**Table 2:** Odds ratios of metabolic factors for ASCVD

	B	SE	Wald	OR	95% CI	P value
Age (per 10 years)	0.595	0.187	10.141	1.814	1.257–2.616	0.001
Sex (f/m)	0.679	0.302	5.053	1.971	1.091–3.562	0.025
Smoking (y/n)	− 0.649	0.351	3.423	0.522	0.263–1.039	0.064
Alcohol (y/n)	− 0.822	0.350	5.526	0.440	0.222–0.872	0.019
High education (y/n)	0.007	0.561	0.000	1.007	0.335–3.026	0.989
Physical activity (y/n)	0.024	0.327	0.005	1.024	0.539–1.946	0.942
Family history of ASCVD (y/n)	0.615	0.272	5.103	1.850	1.085–3.155	0.024
Central obesity (y/n)	0.677	0.351	3.723	1.967	0.989–3.913	0.054
General obesity						
Overweight (y/n)	− 0.111	0.334	0.110	0.895	0.466–1.721	0.740
Obesity (y/n)	− 0.174	0.363	0.228	0.841	0.412–1.714	0.633
Duration of DM (y/n)	0.031	0.039	0.661	1.032	0.957–1.113	0.416
FPG (per 1 mmol/l)	0.158	0.090	3.068	1.171	0.981–1.396	0.080
HbA1c (per 1 %)	0.181	0.068	7.174	1.198	1.050–1.368	0.007
Hypertension (y/n)	0.311	0.343	0.824	1.365	0.697–2.674	0.364
SBP (per 10 mmHg)	0.051	0.066	0.597	1.052	0.925–1.197	0.440
DBP (per 5 mmHg)	− 0.037	0.060	0.368	0.964	0.857–1.085	0.544
TC (≥ 4.68 mmol/l)	− 0.080	0.302	0.071	0.923	0.510–1.668	0.790
TG (≥ 1.70 mmol/l)	− 0.029	0.274	0.011	0.971	0.568–1.660	0.915
LDL-C (≥ 2.60 mmol/l)	0.622	0.362	2.959	1.864	0.917–3.788	0.085
HDL-C (m ≤ 1.00 mmol/l; f ≤ 1.30 mmol/l)	0.217	0.323	0.453	1.243	0.660–2.341	0.501
eGFR (per 10 ml/min/1.73 m^2^)	− 0.105	0.043	5.910	0.900	0.827–0.980	0.015

Binary univariate logistic regression was conducted to assess the association of ASCVD with different variables using the Entry method; odds ratios (ORs) and the 95% confidence intervals (CIs) given

## Discussion

In this study, the prevalence of ASCVD was 15.29% in T2DM patients. Age, female sex, the family history of ASCVD and hyperglycemia were risk factors of ASCVD, similar to previous research results. Coexisting diseases with T2DM, such as hypertension and hyperlipidemia are defined ASCVD risk factors, while DM itself is considered a CAD risk equivalent. ASCVD is the main cause of morbidity and mortality in diabetic patients, contributing to the biggest burden of DM directly or indirectly. The 2007–2008 China National Diabetes and Metabolic Disorders Study reported that the defined total of ASCVD prevalence was 1.44%, including 0.83% of stroke and 0.63% of CHD in Chinese adults over 20 years of age [25]. The Da Qing impaired glucose tolerance (IGT) and Diabetes Study found that the leading cause of death for patients with diabetes is ASCVD after a 23-year follow-up study [26].

It is of great importance for ASCVD to be accurately detected and timely intervened. Due to the fact that the sensory nerve becomes insensitive and even lost in patients with diabetes, symptoms of ASCVD become asymptomatic or atypical. A number of cardiovascular risk assessment tools proposed to assess the risk of developing ASCVD are available, such as Framingham risk score (FRS), Adult Treatment Panel III (ATP-III), EURO—the Systematic Coronary Risk Evaluation (SCORE), Reynolds risk score (RRS), QRISK2 and the Chinese ten-year appraisal method for ischemic cardiovascular disease (ICVD). However, the suitability of these screening assessment tools are limited due to great disparities in terms of ethnicity, geography, environment and lifestyle of patients. Moreover, computed tomography (CT) and angiography can detect the evidence of stenosis or occlusion of vasculars directly, but such clinical applications can result in radiation exposure and sometimes unnecessary invasive diagnosis and treatment. Therefore, it is necessary to look for simple and non-invasive approaches to identify ASCVD early in clinical practices.

In spite of growing controversy, more evidence has revealed a predictive value of microvascular diseases in T2DM on developing ASCVD. In other words, micro- and macrovascular complications of T2DM have a “common soil”. Since the Framingham Heart Study and the Framingham Eye Study had identified the association between DR and the occurrence of cardiovascular events, the importance of DR beyond visual damage was gradually realized by doctors, researchers and the general public. The Atherosclerosis Risk in Communities (ARIC) study also found that DR was associated with an increased risk of ischemic stroke [hazard ratio (HR), 2.34; 95% confidence interval (CI), 1.13 to 4.86] over a follow-up period of 7.8 years on average in middle-aged diabetic patients, independent of other risk factors [14]. Another ARIC study [27] showed that after controlling for traditional cardiovascular risk factors, participants with retinopathy were 2.5 times as likely to develop heart failure (HF) as those without the condition (HR 2.71; 95% CI 1.46 to 5.05). Significant association still existed following further adjustments for glycemic control, carotid atherosclerosis, and serum markers of endothelial dysfunction (HR 2.20, 95% CI 1.08 to 4.47). A recent meta-analysis showed that patients with DR were more liable to develop stroke [risk ratio (RR), 1.74; 95% CI 1.35 to 2.24], compared with those without. Furthermore, DR was associated with an increased risk of HF in patients with DM (RR 2.24; 95% CI 0.98 to 5.14), although marginally [15]. Retinopathy proved to be an independent risk marker for CVD in patients with T2DM [28].

However, there is insufficient evidence showing whether this association can be confirmed from consistent observation in Asian people, especially in Chinese. There exist differences in the epidemiologic and risk associations of ASCVD between white and Asian populations. For this reason, an ethnicity-specific risk model of ASCVD is potentially needed. Furthermore, it is still debatable whether each stage of DR is associated with increased risk of ASCVD. A Finnish report suggested that during a follow-up study lasting seven years, the risk of CHD events was significantly higher than patients without DR, but only in those with PDR at baseline (OR 2.31, 95% CI 1.21 to 4.40) [29]. However, the Japan Diabetes Complications Study (JDCS) [30] which consisted of 2033 patients with T2DM confirmed that the presence of DR was continuously associated with an increased risk of stroke and CHD based on 8 years of follow-up. Additionally, even mild to moderate NPDR triggered a higher risk of CHD (HR 1.69; 95% CI 1.17–2.97) and stroke (HR 2.69; 95% CI 1.03–4.86) after adjusting for traditional cardiovascular risk factors. In our research, those T2DM patients with an eGFR < 60 ml/min/1.73 m^2^ were excluded specifically to avoid the confounding factor of renal insufficiency, which was associated with an increased risk of atherosclerosis. We found that the presence of DR was significantly associated with an increased risk of ASCVD in Chinese with T2DM. Furthermore, the incidence of ASCVD increased along with the severity of DR. After adjustment for age, sex and other traditional risk factors, PDR rather than NPDR was significantly associated with and an independent risk factor for ASCVD. In the presence of PDR, risk of ASCVD was sevenfold in T2DM patients and PDR offered risk information beyond that provided by those established risk factors.

DR is closely related to ASCVD in epidemiology, and similar pathophysiological processes may also contribute to DR and diabetes-accelerated atherosclerosis. To elaborate, traditional risk factors for ASCVD, such as hypertension, hyperlipidemia, duration of hyperglycemia, magnitude of glycemic control and metabolic syndrome (MetS) are well-known for the progression and development of DR [7, 31]. Secondly, neovascularization (retinal angiogenesis) is a typical characteristic of PDR, while angiogenesis is also a common feature observed in advanced atherosclerotic lesions [32, 33]. Early pathological changes of macro- and microvascular have similarities, and chances are microcirculation change can quicken macrovascular lesions. In addition, increasing evidence indicates that unifying mechanisms play a major role in the pathogenesis of diabetic macrovascular, as well as microvascular, complications [34]. These mechanisms include hexosamine pathway, activation of protein kinase C, oxidative stress, pathological effects of the renin–angiotensin–aldosterone system (RAAS), advanced glycation end product (AGE) formation, inflammation and modification of circulating macromolecules, etc., which may influence not only the development of DR, but also the progress of atherosclerosis [35].

As mentioned above, we have elaborated on the usefulness of a simple and non-invasive evaluation of the ASCVD risk in diabetic patients through the research of DR. However, thanks to recent deployment and development of digital technologies, telemedicine has been increasingly applied in many clinical areas such as cardiovascular diseases and diabetes, alongside all its chronic complications, thus assuming an important role as a tool for a diagnosis of DR even at a great distance from doctors or specialist centers [36, 37].

Our study has several limitations that must be taken into account. First of all, it may have unpredictable selection bias. Secondly, ASCVD patients in this article are confirmed cases by hospitals, which means that some potential ASCVD patients may be missed. This might lead to an underestimation of the prevalence of ASCVD in T2DM patients. Thirdly, as these associations are cross-sectional, the study design is incapable of estimating a causal relationship directly; therefore, our findings may suggest that PDR is an indicator, but not predictor of ASCVD. Furthermore, participants are small in number for a cross-sectional study. The adverse effects of DR on ASCVD in Chinese population should be confirmed further in a larger cohort study with a broader spectrum of potential confounding factors. However, we have no reasons to believe these would substantially bias the associations reported herein.

In conclusion, all our data confirm that DR associates strongly with ASCVD in the Chinese population with T2DM. With the severity of DR increasing, the risk of ASCVD also increases. After adjustment for traditional risk factors, PDR is an independent risk marker for ASCVD. Retinal blood vessel is the only microvasculature that can be directly observed, and it would definitely be intriguing to assess whether the retinal microvasculature can be used as a ‘window’ into the state of the cardiovascular complications in patients with diabetes. DR is not only one of the most common microvascular complications of diabetes, but also a “warning sign” for ASCVD. Based on these research findings, we hope that clinicians pay more attention to systemic vascular risk of the patients with DR, especially PDR, and suggest incorporating PDR to clinical cardiovascular risk stratification in patients with diabetes.

## Data Availability

The data used to support the findings of this study are available from the corresponding author upon request.

## Abbreviations

ASCVD: Atherosclerosis cardiovascular disease
DR: Diabetic retinopathy
NDR: No DR
NPDR: Non-proliferative diabetic retinopathy
PDR: Proliferative diabetic retinopathy
DM: Diabetes mellitus
BMI: Body mass index
WC: Waist circumference
FPG: Fasting plasma glucose
HbA1c: Glycated haemoglobin
SBP: Systolic blood pressure
DBP: Diastolic blood pressure
TC: Total cholesterol
TG: Triglycerides
HDL-C: High-density lipoprotein
LDL-C: Low-density lipoprotein cholesterol
eGFR: Calculated glomerular filtration rate
OR: Odds ratio
CI: Confidence interval
